# Why do some women still prefer traditional birth attendants and home delivery?: a qualitative study on delivery care services in West Java Province, Indonesia

**DOI:** 10.1186/1471-2393-10-43

**Published:** 2010-08-11

**Authors:** Christiana R Titaley, Cynthia L Hunter, Michael J Dibley, Peter Heywood

**Affiliations:** 1 Sydney School of Public Health, Edward Ford Building (A27), University of Sydney, NSW 2006, Australia; 2 Menzies Centre for Health Policy, University of Sydney, NSW 2006, Australia

## Abstract

**Background:**

Trained birth attendants at delivery are important for preventing both maternal and newborn deaths. West Java is one of the provinces on Java Island, Indonesia, where many women still deliver at home and without the assistance of trained birth attendants. This study aims to explore the perspectives of community members and health workers about the use of delivery care services in six villages of West Java Province.

**Methods:**

A qualitative study using focus group discussions (FGDs) and in-depth interviews was conducted in six villages of three districts in West Java Province from March to July 2009. Twenty FGDs and 165 in-depth interviews were conducted involving a total of 295 participants representing mothers, fathers, health care providers, traditional birth attendants and community leaders. The FGD and in-depth interview guidelines included reasons for using a trained or a traditional birth attendant and reasons for having a home or an institutional delivery.

**Results:**

The use of traditional birth attendants and home delivery were preferable for some community members despite the availability of the village midwife in the village. Physical distance and financial limitations were two major constraints that prevented community members from accessing and using trained attendants and institutional deliveries. A number of respondents reported that trained delivery attendants or an institutional delivery were only aimed at women who experienced obstetric complications. The limited availability of health care providers was reported by residents in remote areas. In these settings the village midwife, who was sometimes the only health care provider, frequently travelled out of the village. The community perceived the role of both village midwives and traditional birth attendants as essential for providing maternal and health care services.

**Conclusions:**

A comprehensive strategy to increase the availability, accessibility, and affordability of delivery care services should be considered in these West Java areas. Health education strategies are required to increase community awareness about the importance of health services along with the existing financing mechanisms for the poor communities. Public health strategies involving traditional birth attendants will be beneficial particularly in remote areas where their services are highly utilized.

## Background

Each year around four million newborns die in the first week of life, worldwide [1,2], and an estimated 529,000 mothers die due to pregnancy-related causes [2,3]. In low and middle-income countries many deliveries still occur at home and without the assistance of trained attendants [4-7]. This has generated serious concern, since women who develop life-threatening complications during pregnancy and delivery require appropriate and accessible care. A recent review reported that around 20-30% of neonatal mortality could be reduced by implementing skilled birth care services [8].

The effort to increase access to trained birth attendants was initiated by the World Health Organization in 1987 in Nairobi, Kenya, through the launching of the Safe Motherhood Initiative, aimed at ensuring women have a safe pregnancy and childbirth [9,10]. Attention to maternal health was demonstrated in 2000 when 147 heads of state and government and 189 nations in total signed the Millennium Declaration, in which the proportion of births assisted by trained birth attendants became an important indicator to measure the progress of improving maternal health (Millennium Development Goal 5) [11,12].

In 1989 the Indonesian Government embraced the concept of the Safe Motherhood Initiative through the implementation of the "Village Midwife" program. This program aimed to place one midwife in every village to ensure a safe pregnancy and delivery for all pregnant women [13-15]. In its initial phase, nurses were trained in a one-year midwifery program to qualify them to become a village midwife. Later, as the pre-services training was claimed to be inadequate, an additional two-week in-service training was carried out for village midwives in the form of classroom-based training as well as clinical training for the management of normal delivery and life-saving skills [13,15,16]. By 1996, more than 50,000 village midwives had been placed in villages in Indonesia, and the one-year pre-service training was then replaced by a three-year specialist program for high school graduates [13,17]. Between 1970 and the early 1990s, health personnel were employed in the public sector, and with the implementation of the contract scheme, doctors and midwives, not including nurses, worked for a prescribed time period of around three years for the government and then proceeded to either a private practice or a specialist training [18]. It has been observed that the distinction between private and public clinical practice in Indonesia remains unclear [19]. Midwives, including village midwives, employed by the state can also charge private fees for the same services, except for services delivered at health centres.

To encourage the community to contribute to help pregnant women in their own society, in 1998 Indonesia initiated the *Siaga *(alert) program [20]. The *Desa Siaga *(village alert) program embraces the safe motherhood concepts through including community support for pregnant mothers by arranging transport, funds, and access to blood donations. This scheme helps those who have limited financial resources to access health professionals' services through a communal financing mechanism, such as the pregnant mother saving scheme or *Tabulin *(*Tabungan Ibu Bersalin*), and the social funds for pregnant women or *Dasolin *(*Dana Sosial Ibu Bersalin*) [20].

In 2007 a partnership initiative was put forward by involving village midwives and traditional birth attendants through the 'Improving Maternal Health in Indonesia' program [21]. Under this scheme the midwives and traditional birth attendants were expected to work together. The traditional birth attendants could continue to provide services including herbal drinks or post-delivery care, whereas all medical treatment was to be provided by midwives [21].

The Indonesian government committed to providing universal health insurance through a mandatory public health insurance scheme called the Health Insurance for the Poor Population or *Asuransi Kesehatan Masyarakat Miskin (Askeskin)*, as the initial phase of universal coverage in 2004 [22,23]. This scheme evolved into a Health Insurance Scheme for the Population, called *Jaminan Kesehatan Masyarakat (Jamkesmas) *covering more than 76 million poor and near poor populations [22]. This scheme aims to provide free health care services, including antenatal, delivery, or postnatal care services for mothers and infants [23].

The improvement of maternal health care services in Indonesia has been demonstrated by the increased percentage of deliveries assisted by trained delivery attendants - from 43% in 1997 to 79% in 2007 [24,25]. However, the 2007 Indonesia Demographic and Health Survey still reported a large percentage of home deliveries (53%), although the percentage has decreased substantially over the last decade (73% in 1997) [24,25]. The survey also found that 79% of institutional deliveries took place in private facilities such as hospitals, clinics, or private practices of midwives [25]. Although the Indonesian Ministry of Health set a target to achieve 90% deliveries attended by trained delivery attendants by the year 2010, the percentages of home deliveries and deliveries assisted by traditional birth attendants varies widely across provinces in Indonesia.

West Java is one province on Java Island with a high percentage of utilization of traditional birth attendants (30%) and home deliveries (55%) [25]. Although health facilities and health professionals in West Java are available at the village level [18,26], the percentage of deliveries assisted by traditional birth attendants and home deliveries was higher compared to East Java Province, where the percentage of deliveries assisted by traditional birth attendants was only 22% and home deliveries was 32%; or in Central Java where the percentages were 17% and 46%, respectively [25]. These findings indicate the importance of developing strategies at the local level to increase the utilization of delivery care services in this region.

This project is part of a larger study aimed at exploring community members' and health workers' perspectives about antenatal, delivery and postnatal care services in West Java. We present here the results from the analyses of the perspectives of community and health care workers about delivery care services in three districts of West Java. We explored the reasons community members used traditional birth attendants and why they preferred home delivery.

## Methods

### Sampling methods and study sites

This study was conducted from March to July 2009 in West Java Province, the most populous province in Indonesia with more than 39 million people living in 17 districts and nine municipalities [27]. The majority of the population is from the Sundanese ethnic group who speak Sundanese language. Agriculture and industrial production are the main source of livelihoods for the people in this area.

Using purposive sampling methods, three districts with a low use rate of trained delivery attendants were selected, namely Garut, Ciamis, and Sukabumi [28,29]. In 2008, the population in Garut district was 2,481,471 [30]; in Ciamis was 1,538,469 [31] and in Sukabumi was 2,405,777 [32]. The proportion of deliveries assisted by trained delivery attendants in Garut, Ciamis, and Sukabumi districts was 53%, 67% and 66%, respectively [30-32].

The selection of villages was conducted after consultations between the researchers and the local district health office (*Dinas Kesehatan Kabupaten*) of each district. In all districts, villages in urban areas, as defined by the Statistics Indonesia [33], have better access to health facilities such as health centres and health clinics compared to their rural counterparts. Based on this information, two villages were selected from each district to represent urban and rural areas. The selected villages were Sukarame (urban) and Sukajaya (rural) villages in Garut district; Benteng (urban) and Panyutran (rural) villages in Ciamis district; and Batu Nunggal (urban) and Limus Nunggal (rural) villages in Sukabumi district.

All the selected villages had road access. However, rural villages are located in mountainous areas and had generally poor road conditions. During the rainy season, some parts of the villages were not accessible by car and, therefore, villagers had to either walk or ride motorcycles to reach health care facilities. The majority of people in our study areas worked as manual labourers in agricultural or industrial production.

### Study population

**Figure 1:**
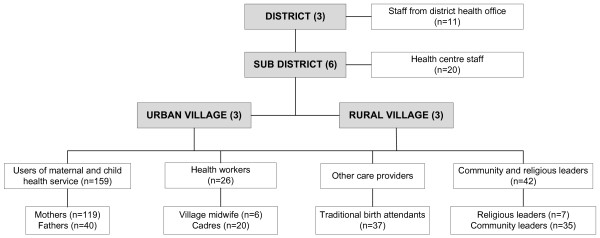
Sampling frame for a qualitative study in West Java, Indonesia

In this study, different groups of participants were selected to provide an overall picture about delivery care services in our study areas from the users' perspectives (i.e. mothers and their husbands, who were assumed to be involved in the decision making process about services), care providers (i.e. health professionals, including midwives and health centre staff and cadres as local community health workers), community leaders, and health authorities (i.e. health office staff). The perspectives of traditional birth attendants were considered important since they reportedly played a prominent role in providing maternal and child services in Indonesia, including in West Java Province [34,35]. A detailed sampling frame is presented in Figure 1.

A total of 295 participants were recruited in this study. These consisted of 119 mothers of children aged more than 40 days to four months, along with 40 fathers, 26 health professionals including 20 health centre staff (doctors, nurses and health centre midwives) and six village midwives, 20 village cadres (local community health workers), 37 traditional birth attendants or *paraji *(in Sundanese language), 42 community and religious leaders, and 11 health office staff.

The recruitment of the mothers, fathers and community leaders was assisted by cadres from each village. Health workers participating in this study were the maternal and child health services providers in health centres at sub-district level, i.e. doctors, nurses and health centre midwives, as well as the care providers at village level, i.e. the village midwife. Information about traditional birth attendants in each village was obtained through the local knowledge of community members and they were individually invited to participate. Several health office staff working in maternal and child health program in each district health office were also recruited.

### Data collection

In this study, two data collection methods were used, focus group discussions (FGDs) and in-depth interviews. Focus group discussions were used to explore information about the social context and issues which might be necessary to further investigate through in-depth interviews. The interaction between participants and hearing from others in a focus group discussion provides a valuable opportunity to show and discuss the differences among participants [36]. In-depth interviews were individually focused to investigate personal perspectives.

Six trained interviewers/facilitators and five field assistants were recruited and trained to collect information in the study areas. A total of 20 FGDs and 165 in-depth interviews were conducted across the six villages. Informed consent, including the consent to use recording devices, was obtained from all respondents. No one refused to give consent in this study. FGDs and in-depth interviews were conducted according to the guidelines as shown in Table 1. Interviews and FGDs were carried out either in Sundanese or Indonesian language.

**Table 1:** Main topics included in the guidelines used for focus group discussions and in-depth interviews

Category of participants	Topics
**Mothers or fathers**:	
**a. Using trained delivery attendants at childbirth**	The use of trained attendants' services
	Obstacles when using trained attendants' services
	History of delivery
**b. Using traditional birth attendants at childbirth**	The use of traditional birth attendants' services
	Obstacles when using trained attendants' services
	History of delivery (including the services provided by the delivery attendants)
**c. Having an institutional delivery**	Reasons for facility-based delivery services
	Obstacles when accessing a health care facility
**d. Having a home delivery**	Reasons for a delivery outside a health care facility
	Obstacles when accessing a health care facility

**Health care providers**	Content of delivery care services provided
	The implementation of delivery care services in the community
	Obstacles when delivering health care services
	Maternal and child health programs available in the community
	Community response towards maternal and child health programs

**Traditional birth attendants**	Type of services provided during pregnancy
	Practice of partnership

**Community and religious leaders**	Perception about maternal and health care services
	Perception about different care providers
	Obstacles using different care providers
	Maternal and chid health programs in the community

**Staff from district health office**	Maternal and child health programs
	Community response towards maternal and child health program
	Obstacles during implementation of maternal and child health programs

**All participants**	Family support and decision making on health services during delivery
	Traditional practices and beliefs during delivery
	Fee for services used/provided

In each village at least two FGDs were carried out for (1) women assisted by trained attendants during delivery, and (2) women assisted by traditional birth attendants. In some villages, additional FGDs were conducted for traditional birth attendants if there were more than five participants. If fewer participants were available, they were invited only to in-depth interviews. In a village in Ciamis district, an additional FGD was conducted for community leaders who were responsible for the *Desa Siaga *program since this was the only village in which the program had been successfully conducted. On average, each FGD consisted of around seven participants, in addition to one FGD leader and one observer/assistant. All discussions and interviews were audio recorded. FGDs were conducted either at the community hall or the respondents' house.

In-depth interviews were conducted within a confidential setting, usually at the interviewee's house. At least two respondents from each category (see Figure 1) were interviewed by trained interviewers, and all the interviews were audio-recorded. No activity was held in health care-related institutions to avoid any hesitation from respondents who might never have had contact with health care services or personnel.

At the end of each activity, the interviewer or FGD facilitator was requested to fill in an evaluation form to help them evaluate the process and the content of their interviews and discussions. This activity would help them to improve or make adjustments in their next activity. One assistant, a Sundanese speaker, was assigned to each interviewer or FGD facilitator. They acted as an interpreter if respondents could not speak Indonesian language as well as an observer during FGDs and in-depth interviews. A cash payment of 50,000 Indonesian Rupiah (~USD 5) was paid to participants to cover their out-of-pocket expenditure. Information leaflets on maternal and child health care were provided to the mothers, fathers, and traditional birth attendants at the end of each activity.

### Framework and definitions

**Figure 2:**
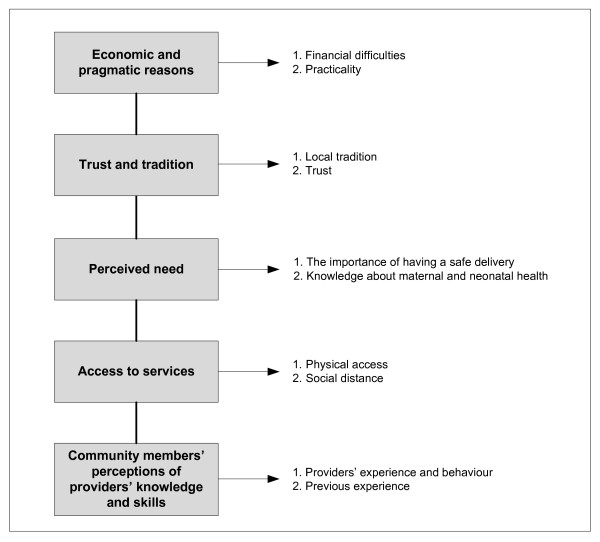
Factors affecting women's decision to use delivery care services

A guideline in the analysis was based on a method previously described in a study of maternal mortality [37]. Factors affecting the decision to select delivery care attendant and place of delivery were divided into five main groups: economic and pragmatic, trust and tradition, perceived need, access to services and community members' perceptions of providers' expertise (Figure 2).

The following standard definitions of delivery attendants based on the Indonesian Ministry of Health and the World Health Organization (WHO) were used. The focus of delivery attendants discussed in this paper is village midwives and traditional birth attendants. Village midwife is defined as "a midwife placed in a village to increase the quality and the coverage of health services of a health centre, with service areas of one or two villages. The village midwife has a role to conduct health services based on the competency and resources in particular delivery services, maternal and child health, and oversee the community in conducting five programs of integrated health posts, which are maternal and children health, family planning, nutrition, immunization, and management of diarrhoea and ARI that includes health promotion" [38]. The village midwife referred to in this study is different from the village midwife referred to in an earlier study in West Java, which identified village midwives as traditional birth attendants who had been trained for some basic biomedical procedures [34].

Traditional birth attendants are defined by the WHO as "a person who assists the mother during childbirth and who initially acquired her skills by delivering babies herself or through apprenticeship to other traditional birth attendants" [39]. A trained traditional birth attendant is someone who "has received a short course of training through the modern health care sector to upgrade her skills. The period of actual training is normally not more than one month, although this may be spread over a long time" [39].

### Data analysis

Each participant was de-identified in the data analysis phase and all data was entered into a password-protected computer owned by the researcher. All the audio-recorded interviews and FGD were transcribed in Indonesian language by the research assistants. The transcriptions were cross-checked with the recordings by the research team and then exported to NVivo 8 (qualitative data analysis software). A content and thematic analysis [36,40,41] was conducted in the Indonesian language by the lead researcher (CRT). For each transcription, issues relating to the study aims were identified and coded without predefined categories. After the completion of the coding process, themes were developed and classified, guided by the framework previously described. A triangulation of data sources and methods [36,40,41] was employed, comparing information from different sources (different categories of respondents), different methods (in-depth interviews and FGDs) using multiple interviewers.

### Ethical clearance

Ethical clearance was obtained from the Human Research Ethics Committee (HREC) at the University of Sydney, Australia and from the Ethical Research Commission National Institute of Health Research & Development (NIHRD), Ministry of Health Republic of Indonesia.

## Results

Our data exposed a range of issues regarding the use of delivery care services in six villages of West Java Province. Five major topics emerged: (1) Reasons for using the services of traditional birth attendants at childbirth; (2) Reasons for having a home delivery; (3) Reasons for using trained delivery attendants and institutional delivery; (4) The partnership practice between the midwife and traditional birth attendants; and (5) Community perceptions about the village midwife and traditional birth attendant. Topics (1) and (2) are discussed separately since they will provide more information about why some community members still preferred traditional birth attendants and/or home delivery services. A woman might be assisted by trained delivery attendants but preferred to deliver at home.

### Reasons for using the service of traditional birth attendants

From 119 mothers participating in this study, more than 40% used traditional birth attendants at childbirth. The participants' reasons for using the services of traditional birth attendants can be classified into five main categories, economic and pragmatic, trust and tradition, perceived need, access to services and community members' perceptions of care providers' knowledge and skills.

#### Economic and pragmatic reasons

Cost was one of the main reasons stated by participants in all villages for using the services of traditional birth attendants. The average delivery cost for a midwife of IDR 350,000 (~USD 35) was perceived as unaffordable by some community members. In addition, the flexibility of the payment method for traditional birth attendants was more convenient.

*We don't have much money. We need to pay around 400 *[400,000~USD 40] *for a village midwife. For a traditional birth attendant... we can pay around 100 *[100,000~USD 10]*. Just depends on how much we have. We can even pay them by instalments. ***(Focus group discussion with mothers, Sukarame, Garut)**

*I'm struggling with my daily expenses, how can you expect me to pay a village midwife*? **(In-depth interview with a mother, Sukajaya, Garut)**

Even though some community members had *Jamkesmas *cards to enable them to access free health care services, the services of traditional birth attendants were still preferred.

*There are so many people seeking care from traditional birth attendants. We have already explained that if you have Jamkesmas, you do not have to pay anything to use midwives' services. But then they said they were still afraid that they would be required to pay. One day they also said they were ashamed of using the midwives services without paying anything. ***(In-depth interview with a cadre, Sukarame, Garut)**

Furthermore, misunderstanding about the eligibility of *Jamkesmas *was also found in the community.

*Jamkesmas is only used for the village midwife service. Other than that *[e.g. private midwife] *you cannot use it. ***(In-depth interview with a cadre, Batu Nunggal, Sukabumi)**

*People were afraid that they will be neglected *[by a health provider] *if they used Jamkesmas*. **(In-depth interview with a community leader, Panyutran, Ciamis)**

#### Socio-cultural - Trust

Our study found that being part of the community, speaking the local language, living in the community and sharing the same culture meant that traditional birth attendants have developed the feeling of trust in the community.

*Traditional birth attendants are much closer to the community. They have been treated as a respected person by the villagers. Sometimes the village midwife could not adapt really well with the surroundings; whereas for traditional birth attendants, they grow together with the community. Psychologically, they trust the traditional birth attendants more. ***(In-depth interview with health office staff, Garut**)

#### Socio-cultural - Tradition

Another factor that influenced the use of traditional birth attendants was being told by other family members such as the older sister, parents, or husbands to use their services. A long-time tradition in the community of using the service of traditional birth attendants, who had been the only delivery service providers for many years before the National Health System started, was also mentioned as a reason for community members to use their services during childbirth.

#### Perceived need

Some participants argued that the services of a health professional (a village midwife) are required only for those experiencing obstetric complications. Some community members stated that the midwife's services would be sought only if the condition could not be handled by the traditional birth attendant.

*In this village, the first assistant from whom we seek care is the traditional birth attendant. If the delivery starts to be complicated, we then call midwives. ***(Focus group discussion with fathers, Limus Nunggal, Sukabumi)**

*They think deliveries assisted by midwives are only for those with delivery complications... If traditional birth attendants cannot manage the deliveries, then they would call us. Otherwise, calling midwives is unnecessary. *(**In-depth interview with a village midwife, Sukajaya, Garut)**

*There are many cases like this, for example an obstructed labour with excessive bleeding, or retained placenta cases. They just wait until the traditional birth attendants could no longer manage it. Sometimes we arrived late and the mother already had severe oedema and was in a very weak condition. ***(In-depth interview with a health centre midwife, Ciamis)**

#### Access to services

Three reasons related to the issues of accessibility to health care services were physical distance, time constraints, and the availability of a health care provider. In rural areas there was better access to the traditional birth attendants compared to the village midwife. Some rural villages had more than ten traditional birth attendants compared with only one village midwife.

*The village midwife service is ... too far away. It is impossible to contact the health personnel at ten o'clock at night. It is impossible. Better to use the services of traditional birth attendants. ***(In-depth interview with a religious leader, Sukajaya, Garut)**

*At night time, the major problem is we have a poor road condition...especially during the rainy season. It is difficult to find the midwife by yourself. ***(In-depth interview with a father, Panyutran, Ciamis)**

*The delivery was at night and it was an emergency. The baby was already out. The closest was the traditional birth attendant so I just ran to ask for her help. ***(In-depth interview with a father, Sukajaya, Garut)**

*Sometimes the midwife was not at home. Sometimes her husband also did not allow her to go out at night. He was afraid something bad might happen along the way. ***(Focus group discussion with mothers, Sukajaya, Garut)**

*The traditional birth attendants live closer than the midwife. There is only one village midwife for the whole village, so she also has limited ability to serve the whole community, maybe because she is tired, or needs to travel out of the village. ***(In-depth interview with a midwife coordinator, Sukaresmi, Garut)**

The social distance between the village midwife and the community was also an issue. Some community members were hesitant to seek a midwife's services even if they had received a *Jamkesmas *card that makes them eligible to use services for free.

*The midwife assured me not to worry since I have Jamkesmas. But I feel ashamed for waking her up in the middle of the night. For me it is fine with traditional birth attendants. We are used to them. Yet for the midwife, I feel ashamed for not having any money to pay her. ***(In-depth interview with a mother, Sukarame, Garut)**

#### Community members' perceptions of care providers' knowledge and skills

For some community members, village midwives were also perceived as too young and inexperienced; whereas traditional birth attendants were more mature, patient and caring compared with the midwife.

*They say the traditional birth attendants are more patient. They gently touch your stomach and do not easily feel upset. This attitude is different from midwives. Sometimes after the physical examination, the midwife leaves if she thinks it is not the time for delivery yet. In contrast, the traditional birth attendant will wait patiently and accompany the woman all along. ***(In-depth interview with a traditional birth attendant, Batu Nunggal, Sukabumi)**

### Reasons for a home delivery

Almost 80% of mothers participating in this study delivered at home. Similar reasons were given by the participants when asked about home delivery to the ones given for use of a traditional birth attendant at childbirth.

#### Economic reasons

Particularly among those who did not have the *Jamkesmas*, cost was one of the major reasons for not having an institutional delivery.

*You need around IDR 2 million *[~USD 200] *to deliver in the hospital. With traditional birth attendants, even 50,000 *[~USD 5] *is fine. Our husbands only work as manual labours. Where can we get the money to pay the midwife or to deliver in the hospital? ***(Focus group discussion with mothers, Limus Nunggal, Sukabumi)**

*I prayed that day, "Lord please do not let my wife deliver our baby in the hospital. We do not have any money." ***(Focus group discussion with fathers, Benteng, Ciamis)**

#### Perceived need

There is a perception that delivery is a natural rite of passage for women, and thereby home delivery is preferred unless complications occur or someone tells them to deliver at health facilities.

*If there were some problems then the mother will be brought to the health centre, otherwise she will deliver at home. ***(In-depth interview with a community leader, Panyutran, Ciamis)**

#### Access to services

In addition to the costs, physical distance was an issue for community members living far away from the health facilities and, therefore, home delivery was preferred.

*Maybe distance is an obstacle in addition to the costs. You need to ride a motorcycle to reach the health facility. So people prefer having the health professional to come to their houses, especially at night time when it is hard to get transport. ***(In-depth interview with the head of health centre, Garut)**

*The midwife's place is too far away. It is too far to go to deliver my baby. ***(Focus group discussion with mothers, Sukajaya, Garut)**

#### Convenience

The convenience of home delivery related to the responsibilities pregnant women felt towards other family members.

*If we delivered at the midwife's place, we automatically needed to stay overnight. Maybe for one or two days. I have two young children at home, 4 and 2 years old. Who will take care of them? I look at it that way. So I better deliver at home. ***(Focus group discussion with mothers, Cibadak, Sukabumi)**

*They just said they do not want to bother anyone. Delivery in the midwife's place means someone needs to go and accompany you. At home they can just wait for the delivery time while doing some household chores. ***(In-depth interview with a cadre, Sukarame, Garut)**

### Reasons for using trained delivery attendants and institutional delivery

Our study found that delivery complications at childbirth were a main reason for using the service of health workers at childbirth (55% of the mother respondents) and for having institutional delivery (20% of the mother respondents).

*Those who seek midwives services are usually those who have difficulties delivering their babies. ***(In-depth interview with a community leader, Panyutran, Ciamis)**

*If there is no problem we use traditional birth attendants. But if it looks like the traditional birth attendant could not manage it, we will call a midwife. ***(In-depth interview with a mother, Cibadak, Sukabumi)**

*We called the village midwife, and she asked us to bring me to her place. But then she was not able to help me because I had hypertension. So they brought me to the hospital. ***(Focus group discussion with mothers, Sukarame, Garut)**

Some respondents stated that the competency of midwives and better equipment were amongst the reasons for community members to use their childbirth services. Furthermore, advice from midwives was another reason for mothers to have an institutional delivery.

*It is better to use the midwife service. It is a guaranteed treatment. ***(In-depth interview with a father, Sukarame, Garut)**

*The traditional birth attendant does not have a complete range of equipment in case something happens. If we have the midwife, we do not have to go anywhere anymore. So we just go straight to the midwife. ***(Focus group discussion with mothers, Limus Nunggal, Sukabumi)**

*Because I felt pain so I checked with the midwife. She said I was in labour and I was not allowed to go home. ***(Focus group discussion with mothers, Sukarame, Garut)**

### The partnership practice between midwife and traditional birth attendants

Health professionals in all six villages were aware of the partnership programs between midwives, traditional birth attendants and cadres. However, the implementation varied across villages. In one village in Ciamis district, the partnership was successfully endorsed by the *Desa Siaga *program engaging the village midwife, traditional birth attendants and cadres. In fact, in this village a penalty was given by the *Desa Siaga *officers (mainly the village community leaders from the village) to the delivery attendant if both the village midwife and traditional birth attendants were not present at childbirth (see quotation below).

*The delivery should be assisted by both a traditional birth attendant and a midwife. There was an agreement in all sub-districts, if I am not mistaken. You have to pay 500,000 *[~USD 50]*, divided between traditional birth attendants and village midwife... a penalty will be applied if for example the traditional birth attendant was the only attendant at delivery. ***(In-depth interview with a cadre, Benteng, Ciamis)**

In other villages, the partnership program was not conducted according to the guidelines.

*We have disseminated the partnership program to the community... But we still have some problems with traditional birth attendants... maybe the remuneration was too little. We have even had the meeting together with the doctor *[from the health centre]*, and sub-district office staff... but still it is not working. ***(In-depth interview with a cadre, Sukajaya, Garut)**

*The traditional birth attendants have been asked to work together. But it is difficult. They used to say that the baby is already out *[they did not call village midwife]. **(In-depth interview with a village midwife, Sukarame, Garut)**

The implementation of the partnership program was also hindered by the fact that traditional birth attendants in some villages preferred to work independently without the assistance of health professionals, unless perceived necessary.

*If it could not be managed, we then called the midwife. Otherwise, we will just manage it ourselves. ***(Focus group discussion with traditional birth attendants, Limus Nunggal, Sukabumi)**

*It* [partnership program] *is difficult here. If something happened then they called the midwife. ***(In-depth interview with a village midwife, Limus Nunggal, Sukabumi)**

### Community perceptions of village midwife and traditional birth attendants

The data provided positive feedback about the role of village midwives in the community. They were perceived as diligent, kind, friendly, responsive, alert and willing to provide health services. Nevertheless, the role of traditional birth attendants was considered essential especially in remote areas. Having both a midwife and a traditional birth attendant present at a delivery was perceived important so that the tasks and responsibilities could be shared together.

*We prefer having both of them. Before calling the midwife, we called the traditional birth attendant. The traditional birth attendant will gently touch the mother, and have some special prayers for that. For us village people, that is helpful. The midwife can take care of the child; the traditional birth attendant can look after the mother. If only a midwife is available, she might not be able to handle everything. There should be both of them. ***(Focus group discussion with fathers, Benteng, Ciamis)**

## Discussion

### Main findings

The use of traditional birth attendants and home delivery were preferable for some community members in spite of the availability of a village midwife in the village. Some major factors for the use of both traditional birth attendants and home delivery were the economic and pragmatic reasons, since delivery costs with a midwife or at health care facility were perceived unaffordable. This was aggravated by the low economic status of the community members in addition to the embarrassment and misunderstanding of the *Jamkesmas *scheme. Other reasons found were the trust and tradition that traditional birth attendants engendered; they shared the same culture and were long-serving members of the community. The services of trained birth attendants during childbirth or an institutional delivery were perceived important by some community members only during obstetric complications. Furthermore, difficult access to health care personnel and facilities was amongst the major reasons for preferring traditional birth attendants and home delivery. The social distance between the community and village midwife also emerged as an issue. Our study found that home delivery was considered more convenient for some women because of their responsibilities to children or other household members. The implementation of the partnership program between village midwives and traditional birth attendants varied across villages. The roles of village midwives and traditional birth attendants were perceived vital, particularly in rural areas where health care services were sub-optimal.

### Issues affecting delivery services utilization

Our study demonstrated that for some community members, assistance by the traditional birth attendants and home delivery were preferable, although data from the 2007 IDHS showed that the use of traditional birth attendants and home delivery in Indonesia has been decreasing over the last decade [25].

Our findings follow other studies [37,42,43], which demonstrated that poverty is a major factor influencing people's decision-making about health services. An analysis of different IDHS data also found a significant association between wealth index and the use of health care services [44,45]. Since most of the population in our study areas worked as manual labourers, such as farmers or industrial workers, and had a low income per capita [30-32], the midwife's services or an institutional delivery were more likely to be unaffordable. A deprived financial situation is often linked to low education levels, which affects one's ability to seek the most appropriate health care services [4,46].

The provision of *Jamkesmas *to the poor and near-poor households was expected to help disadvantaged communities to access health care services without burden to their pocket. At present the poor community's embarrassment at using *Jamkesmas*, which enables them to use health service for free, in addition to misinterpretation and misunderstanding about community members' eligibility for *Jamkesmas*, demonstrate an apparent failure of the Ministry of Health and the local authorities to communicate and explain its use and benefits. As a result, the provision of *Jamkesmas *does not change the health care seeking behaviour of some women from disadvantaged households. This finding shows that health care professionals should be encouraged to use every opportunity to promote the use and the understanding of *Jamkesmas *and its benefits to the community. Monitoring and evaluation strategies at the national and local levels for the distribution mechanisms and the effectiveness of *Jamkesmas *are essential. Strengthening *Desa Siaga *programs is important to help families who have experienced financial difficulties to access health services through the community-based financing mechanism.

Proximity to health care facilities is an underlying issue for selecting delivery health care services, as also shown in previous literature [34,37,43]. Poor road conditions and lack of transportation are associated with increased costs of visits to health care providers. An earlier study from West Java Province mentioned the problem of distance as a reason for a community's use of traditional birth attendants compared to midwives [34]. This may be aggravated by the unwillingness of a midwife to make a long trip at night [34]. Reducing the distance by bringing either the community closer to the services or bringing the services closer to the community will be beneficial. Maternal waiting homes where women at the end of their pregnancies can stay and wait for labour have long been advocated to close the physical distance. However, a recent review reported a wide range of views regarding their effectiveness [47]. Therefore, careful planning that takes into account socio-cultural factors is essential. Women preferred to stay at home for a delivery so that they could take care of family members and manage their daily household chores. This indicates that taking women away from the family, even during labour, might not be the most acceptable and appropriate solution.

The social and psychological distance between community and health professionals is an obstacle in some areas. This can be due to the shortage of health professionals in large and remote areas, a frequently absent midwife, or one who does not live in the village [17,48]. A high rate of absenteeism occurs as village midwives often prefer to live in urban areas. With the current expansion of the private health sector in Indonesia, urban areas are much more appealing for health professionals, including village midwives, to establish a private practice while at the same time also receiving the government subsidy [18]. Different strategies have been implemented aimed at retaining village midwives in the villages [17]. However, working in remote areas carries some concerns for midwives and their families, including professional isolation or the pressure of exclusion from a long-established or traditional community [13,17]. Furthermore, if the midwife who is perceived as young, inexperienced, with no children and lacking maturity, is placed in a rural area then the gap may widen further [48]. This is a real challenge. Partnerships between village midwives, cadres, and the community should be strengthened to overcome these issues.

Our study found a lack of awareness about the importance of trained delivery attendants. For some community members, the assistance of trained delivery attendants during childbirth was perceived as only necessary when obstetric complications occurred. Recognition of the need for health services is important to ensure appropriate health care seeking behaviour. For women, childbirth is often perceived as a normal event and normal work, rather than an event requiring medical attention [7,37]. However, the lack of community members' knowledge about symptoms which require medical care can lead to delays in recognition and treatment of severe complications contributing to maternal deaths [3]. Therefore, health promotion strategies are important to increase community awareness about the importance of trained delivery attendants. This can be through antenatal care services which are effective in increasing the use of trained delivery attendants during childbirth and institutional delivery [49,50].

### The role of traditional birth attendants

Traditional birth attendants have been part of the Indonesian community for a long time, before the Safe Motherhood Initiative was endorsed in Indonesia. This profession has been handed over from one generation to another. Our study showed that their role was still prominent. In all villages, traditional birth attendants outnumbered the village midwives. Considering village midwives' limited ability to reach the community to provide health services due to availability, accessibility and social distance, it was seen as quite acceptable that traditional birth attendants' services would be widely used. Their expertise was valued due to their social and emotional closeness to the community, their long experience in providing services to mothers and infants, and their intimacy with the villagers, which created loyalty and understanding, particularly when other health care services were not accessible. This built the authoritative knowledge conferred on them by the community [51].

In the past, training programs for traditional birth attendants were conducted and free delivery kits were provided. Unfortunately, this program has been phased out following the Ministry of Health recommendation that training for traditional birth attendants should be stopped in areas where a village midwife is available [17]. As a result, several traditional birth attendants, particularly the new ones, whose services are being widely used, have not attended any training programs and their ability to conduct a safe delivery remains uncertain.

Previous trials of delivery and postnatal care services involving traditional birth attendants in India and Pakistan demonstrated that engaging traditional birth attendants in the maternal and child health services had a favourable impact on neonatal and perinatal mortality [52,53]. Discontinuing training for the potential traditional birth attendants who are actually capable to provide appropriate care has been claimed to bring more harm than good [54]. A review of the effectiveness of the training of traditional birth attendants also showed favourable results concerning perinatal and neonatal mortality, although the number of the studies included was considered insufficient [55]. A well designed and coordinated training of traditional birth attendants might provide a favourable result [56]. A review of 15 traditional birth attendants and midwife-based interventions aimed at improving delivery assistance skills and recognition as well as referral of complications, demonstrated that both traditional birth attendants and community-based midwives had a role in reducing the maternal mortality ratio [57]. It is a strategy worth reconsidering in Indonesia, particularly in areas where health care facilities and personnel are still lacking and the utilization of traditional birth attendants is high. Some adjustments to the maternal and child health programs should be conducted, taking into account the traditional values in the community.

### The partnership program

Although the partnership program between trained and traditional birth attendants has been endorsed, the implementation of this program varied across villages. In some areas, traditional birth attendants preferred providing services independently and sought midwifery care only if considered necessary. Consequently, adverse delivery outcomes might occur due to the delay of midwives for obstetric emergency conditions. In one village where the partnership program was successfully endorsed, community participation was the key factor in its success. Initiated by local community leaders through the *Desa Siaga *program, different strategies have been carried out to improve community awareness and utilization of the village midwife in addition to the traditional birth attendant. Efforts to strengthen the partnership program would appear to be a beneficial intervention. Advocacy, dissemination, and monitoring activities should be carried out regularly. Local stakeholders, such as community leaders and traditional birth attendants should be encouraged to develop this program, adjusting and adapting it to local conditions to ensure its sustainability.

Compared to a previous study in West Java [34], a more positive attitude towards health care professionals was found amongst the traditional birth attendants in all of our study areas. Some traditional birth attendants perceived health professionals as partners in providing maternal and child health services, and their willingness to collaborate provides a valuable opportunity for village midwives to reach the local community. The involvement of local stakeholders, including the traditional birth attendants is essential when planning and implementing public health interventions.

With the emergence of the decentralization program, in which the responsibility of health services has been transferred to the district level, stakeholders at the district, sub-district and village level play a vital role in the improvement of maternal and child health services in their areas [58]. The identification of resources and constraints to conduct local-based public health strategies is essential. Efforts to implement different approaches adjusting for local conditions should be encouraged and strengthened to increase service uptake.

### Significance of the study

The results of this qualitative study demonstrate perspectives on delivery care services in six villages of West Java Province. These findings provide local evidence that will assist the government in achieving the target of 90% deliveries assisted by the trained birth attendants and to develop public health strategies for the improvement of maternal and child health services. In general, our findings showed that no magic bullet solution is available to increase the utilization of delivery care services in Indonesia. Since the adoption of the Safe Motherhood Initiative in 1989, various maternal and child health programs have been implemented, however, continuous and rigorous evaluation and monitoring programs are required to assess the effectiveness of each intervention. Community-based education that targets the disadvantaged community is required. Peer-education programs that have been shown to effectively increase the use of health care providers and institutional delivery should be implemented [59]. This strategy will increase community awareness about the importance of maternal and children health.

Using a purposive sampling method the results of this study may not be representative of West Java or the Indonesian population; however, using qualitative methods enabled us to explore and understand the perspectives of community members on delivery care services. Triangulation by using different data collection techniques, i.e. in-depth interview and FGD, along with the use of multiple interviewers and different categories of participants increases the validity of the results [36,41].

This study has a number of limitations. We did not differentiate traditional birth attendants who had received biomedical training from those who had not been trained. Although all of the research assistants speak Sundanese and had a role as an interpreter either for the interviewer or respondents, the language issue might have influenced the interaction process during data collection. However, these limitations are unlikely to influence the validity of the results of our study. Further research to examine the extent to which low utilization of these delivery care services might affect maternal and neonatal death could be conducted.

## Conclusions

Our findings show the importance of adopting a comprehensive approach to increase the availability and accessibility of maternal and child health care services in the community. The under-served community are the poorest population who are in the greatest need. Poverty alleviation strategies will contribute to improving access and utilization of maternal and child health care services. The provision and the maintenance of infrastructure by the local government will improve access to health care services, especially for communities living in remote areas. The evaluation and monitoring programs for the current insurance scheme, *Jamkesmas*, are also important to maximise its benefit among poor communities. Health promotion programs to increase community awareness about safe delivery services will benefit the community.

Strengthening the partnership program between village midwives and traditional birth attendants is recommended because of the frequent use of traditional birth attendants in this area. Training of traditional birth attendants would enable them to up-skill their delivery practice under the supervision of health professionals, especially in rural and remote areas. Additionally, local stakeholders' participation plays a major role in the successful implementation of maternal and child health programs.

## Competing interests

The authors declare that they have no competing interests.

## Authors' contributions

All authors designed the study. CRT conducted data collection. Under the supervision of CLH, CRT conducted data analysis and wrote the first draft of the manuscript. MJD and PH provided data analysis advice and revision of the final manuscript. All authors read, commented on and approved the final manuscript.

## Pre-publication history

The pre-publication history for this paper can be accessed here:

http://www.biomedcentral.com/1471-2393/10/43/prepub
